# Care demand networks in maternity care - an innovative approach exploring the complexity of care demands with routine data: Retrospective observational study

**DOI:** 10.1016/j.ijnsa.2026.100532

**Published:** 2026-04-16

**Authors:** Diana Trutschel, Luisa Eggenschwiler, Niklaus Gygli, Jack Kuipers, Giusi Moffa, Michael Simon

**Affiliations:** a Institute of Nursing Science, University of Basel, Basel, Switzerland; b Chief Nursing Office, University Hospital of Basel, Basel, Switzerland; c Department of Biosystems Science and Engineering, ETH Zürich, Switzerland; d Department of Mathematics and Computer Science, University of Basel, Switzerland

**Keywords:** Delivery of health care, Electronic health records, Maternal health services, Nursing staff, Midwifery, Retrospective studies, Routinely collected health data

## Abstract

**Background:**

Care demand is complex, variable, and intense. Yet, most staffing tools that aim to inform staffing requirements reduce it to overly simplified metrics. Electronic health records contain substantial information that could support decision-making as affected by care demands. Standardized care activity catalogs document nursing interventions and the associated care time spent, offering rich insights into care delivery. However, their combined potential to inform staffing models and for exploring the complexity of care remains untapped. Network analysis allows for the analysis and visualization of critical patterns in care demands.

**Objective:**

This study applies network analysis to explore maternity care demand and illustrates how to describe care demand networks according to network terminology.

**Design:**

Retrospective observational study using routinely collected data.

**Setting(s):**

Maternity units in a Swiss tertiary hospital.

**Participants:**

2 346 maternal case records during the postnatal period.

**Methods:**

A correlation network analysis was conducted using 244 382 recorded care activities, with the minutes associated for each activity and following individual cases summed up over the hospital stay. Pearson correlations between activities were estimated to create a partial-correlation network, while edges with |correlations| ≥ 0.15 were retained. The final undirected, weighted graph was analyzed using standard network metrics to explore the features of care from the network structure.

**Results:**

A total of 113 different care activities were recorded, with an average duration of 3.7 min. The resulting network suggests dense subgroups of correlated care activities in the maternity care process.

**Conclusions:**

This study demonstrated the successful application of network methodology to visualize and enhance the understanding of maternity care using routinely collected data. The network approach can be further explored to understand day-to-day care demands and eventually to predict care demands for upcoming days, which fosters staff rostering based on previous care demands.

**Registration:**

*not registered*

#HealthServicesResearch #RoutineData #Workload

**What is already known**

 

• *Existing estimation tools either classify or summarize care demand*.
• *Network analysis has been shown to help understand complex systems*.
• The potential of recorded care activities *to assess the complexity of care activities is underused*.

**What this paper adds**

 

• *The study shows the potential of network analysis to explore and enhance the understanding of maternity care complexity*.
• *Insights of care networks could be the basis to allow unit managers to adjust care supply as needed*.

Alt-text: Unlabelled box dummy alt text

## 1 Background

### 1.1 Care demand estimation

Griffiths et al. (2020) describe several broad domains of tools that estimate care demand and aim to translate them into staffing requirements. Among them, the timed-task approach involves estimating the care time required to complete specific care tasks for each person receiving care and is particularly relevant to capturing a detailed and precise picture of care demand. However, this approach has not been extensively used in research, possibly due to the effort required to gather such detailed data. Importantly, many healthcare settings already routinely document time-stamped nursing activities in electronic patient record systems, enabling the use of a timed-task approach without additional data collection or documentation burdens. Analyzing the recorded care time allocated to distinct care activities could provide a more comprehensive and accurate picture than care time estimated based on subjective judgments, especially as the former demonstrates the proportion of care time required for different activities. Additionally, such analyses offer valuable insights into the necessity of nursing staff with specialized expertise for performing specific care activities. In general, the method that provides the most precise estimate of care demand should be preferred, especially when it can be implemented using routinely collected data and does not require additional documentation effort from nursing staff. This helps plan the number and mix of caregivers needed, avoiding staff burnout and maintaining safety.

### 1.2 Time-task approaches through electronic health records

Electronic health records in hospitals, especially when available in a structured form, serve as comprehensive repositories of patient data and clinical care with the possibility of secondary use (Hayrinen et al., 2008; Vuokko et al., 2017). Thus, electronic health records contain a significant amount of information to support clinical decision-making processes when suitable data extraction methods are applied. They are therefore a large resource to gain a better understanding of clinical care knowledge (Osheroff et al., 2007; Pereira et al., 2012; Wright et al., 2016; Davis et al., 2023; Yakob et al., 2024). Several nursing classification systems have been developed to document nursing care in electronic health records using standardized nursing terminology, aiming to provide comparable data for further research in nursing context (Cesare and Zega, 2024). The *“Leistungserfassung Pflege” (LEP®) system* refers to the Nursing Interventions Classification System and has been widely incorporated into electronic health record infrastructure. Over 800 healthcare organizations in Germany, Austria, and regions of Italy, as well as >70 % of Swiss hospitals, use this standardized care activity catalog to document care activities. The nursing interventions and the required time are recorded either manually or nowadays largely automatically derived from care documentation in electronic health record systems. Therefore, LEP*®* data essentially represents a *time-task approach*. Using care activities data for a comprehensive illustration of care demand provides a unique opportunity, given the extensive dataset of care activities and care time information available. Despite the richness of this data source providing insights into care delivery, its potential for estimating staffing requirements and unraveling care complexity remains largely untapped.

### 1.3 Exploring the complexity of care demand using network analysis

While understanding the complexity of care demand is essential to adjust staffing appropriately, this can be approached via available computational methods. Such methods can handle complexity instead of reducing it and make critical points visible. The longitudinal recording of a wide range of care activities applied to hospitalized persons can be used as a representation of care demand, comprising several levels of complexity. One specific view of care complexity is on the *correlation between different care activities -* the amount of care time spent on each activity in relation to the care time spent on other activities.

**Fig. 1:**
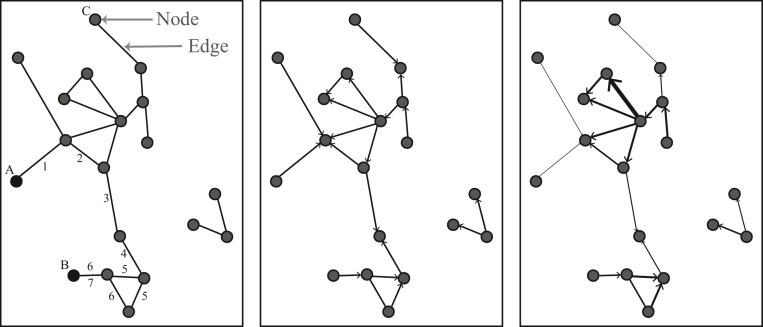
Graphical illustration of several types of networks from left to right: undirected and unweighted network, directed and unweighted network, directed and weighted network. A **node** represents an entity of a system, and the **edges** represent relationships between two entities, where the **direction** (middle) and the **strength** (right) of those relationships could be assigned. Node A and node B can be connected by several **paths**, where the two example paths have lengths (the total number of edges on the path) of 6 and 7. Hence, the **shortest path length** for connecting A and B is 6.

Considering care data as a system of related care activities, *network analysis methodology* could help to explore latent structures in these systems, driven by the data. Particularly, a network can illustrate a complex system through revealing the relationships between system entities based on relationship measures. It allows the *graphical representation of the system* as a network of *nodes*, the entities of the system, and *edges*, representing direct or indirect relationships between two entities (Fig. 1). Depending on the context and the way these *relationships* are expressed, edges can represent different meanings. For instance, in social networks, a connection between two individuals, such as knowing each other, is formulated, whereas in biological networks, it is often the correlation between two units of interest, e.g. in gene-to-gene networks, that may represent their co-expression in cells. Further, the overall structure or the properties of the network intuitively show potential mechanisms within the system. Network analysis then provides a range of quantitative techniques that can summarize a *network's structure* and quantify its *elements' importance*.

The statistical network modeling methodology was initially developed in bioinformatics (Mayer and Hieter, 2000; Skipper, 2005; Fiscon et al., 2018; Sonawane et al., 2019; Silverman et al., 2020), but is also established in social network analysis (Freeman, 2004; Kadushin, 2012; Borgatti, Everett and Johnson, 2018). One prominent example of applying social networks in the healthcare context is to explore complex social relationships or contacts among several actors, e.g. individuals, healthcare teams, or organizations (Espinosa and Clark, 2014; Tasselli, 2014; Sabot et al., 2017; Francis et al., 2024). Although various questions within health services research are suitable to answer by a network approach (Saatchi, Pallotti and Sullivan, 2023), the method is not yet widely used in this field. To our knowledge, no study exists that applies a network analysis to care or similar data, whether to investigate the relationships between care activities or between individuals with similar received care. Moreover, the common way of interpreting underlying network structures to gain system insights may not be applicable here. Consequently, it is essential not only to introduce network terminology but also to adapt the terms to the care context. To better illustrate this, we provide an application example in maternity care, more specifically in the postnatal unit. We chose this example because of its rather homogeneous case mix in one unit, so that the identification of networks should be rather straightforward. With regard to the clinical relevance, it has been shown that care quality in postnatal units is reduced when shifts are understaffed (Turner et al., 2024). This understaffing might occur due to high variability and, so far, missing predictability of care demand (Eggenschwiler et al., 2025).

This methodological article aims to illustrate the *application of network analysis* using care activity data from mothers in hospitals’ *maternity care* as an example of what the method can offer in interpreting received care, and translating the network terminology into terms that make the connection to care. Our motivation for the article is to comprehensively understand care demand through this intuitive visualization method as first step toward exploiting the full potential of network follow-up analysis for staffing adjustments. To achieve the overall aim, the article has three specific objectives: 1) it introduces the network framework and its areas of application, 2) it explains the data, the exact methodological approach used to apply network analysis, and the obtained results, and 3) it discusses the appropriateness of using network analysis to explain the complexity of care demand data and its future potential for answering health service research questions.

## 2 Methods

### 2.1 Network analysis methodology

**Networks** are commonly used to investigate complex systems by representing entities as nodes and their relationships as edges.

**Network methodology** technically distinguishes several *types of networks*: i) directed or undirected, and ii) weighted or unweighted (Fig. 1). All the previous examples from social and biomedical research are types of *undirected graphs,* which means the connections are mutual. In contrast, *directed graphs,* e.g. *Directed Acyclic Graphs,* allow for the illustration of a direction. For example, on social media networks, “following” creates a directional connection from the follower to the followed user. Additionally, *weighted networks* enable us to assign strengths to the relationships between system entities, such as by the size of the edges, if the strength of these relationships can be quantified.

**Network analysis** aims to gain insights into systems by elaborating their structure (Barabási, 2016; Hamilton, Ying and Leskovec, 2018; Hevey, 2018) and help to describe the nature of the network. Therefore, several structural measures (metrics) related to nodes, edges, paths, or the global network topology are commonly used to describe the graphical characteristics of the derived network. This helps to identify the position and importance of system components, and allows for the comparison of several networks by their network characteristics. Since these key metrics are standard definitions (Wasserman and Faust, 1994a; Newman, 2010) but not commonly used in health service research, they are reviewed in more detail below and summarized in Table 1. Node and edge-level centrality metrics include degree and betweenness centralities; network-level structural metrics include distance measures, density, clustering coefficients, and modularity.

**Table 1:** Structural metrics for undirected networks sorted by the network structure units (nodes, edges, network), with the interpretation regarding care activity networks.

Metrices	Definition	Indicates	Interpretation within a Care Activity Network
***Nodes: Vertex represents an entity of the network***			

Node degree (centrality)	The **number** of edges directly linked to the node.	How many relationships are given to an entity? Nodes with a high degree are known as **hubs** (Albert and Barabási, 2002), which indicate central entities within the system (Fig. 2 A1).	How often are care-time allocations for a given care activity associated with care-time allocations for other activities? The application of hub care activities implies that for most of the mothers, many other activities are essentially applied (over the whole stay on the postnatal unit) A care activity for which the time spent providing care is strongly correlated with the time spent on many other activities suggests that it represents a common or routine element of maternity care.
Betweenness (centrality)	How often the node is present within the group of shortest paths in the network in **relative numbers** (calculated as the number of shortest paths in the network that pass through that node divided by the total number of shortest paths).	How often is an entity involved in the relationships present in the system? A node with a high betweenness centrality indicates an involvement in many other relationships, it acts as a bridge or a critical connector between different parts of the network (Fig. 2 A2).	Care activities with high interconnectedness centrality may signify involvement in care procedures including several care components. They may be considered strategic since they may be relevant for the whole care process.

***Edges: Link represents a relationship between two entities of the network***			

Betweenness (centrality)	How often is the edge present within the group of shortest paths in the network in **relative numbers** (calculated as the number of shortest paths in the network that pass through that edge divided by the total number of shortest paths)	How often is a relationship involved in all relationships? An edge with a high betweenness centrality indicates an involvement in many other relationships.	A pair of care activities often applied together during hospital stays and have high interconnectedness centrality may signify involvement in complex care procedures. They may be considered strategic since they are interdependent in managing the whole care process or bridging different types of care (e.g. standard care routines and specialized care).

***Network: A complex system of nodes and edges***			

Diameter	The **longest** geodesic distance in the network.	It is the maximum distance between any two nodes in the network. In smaller networks, the longest shortest path tends to be shorter, due to denser connectivity of the network, in larger networks typically longer, indicating that there are fewer direct paths between distant nodes.	A longer network diameter in a care activity module may indicate a more fragmented or complex care, moreover a variability in individual care pathways, which means that not all individuals require the same combinations of activities, but those are indirectly connected.
Characteristic/ Average path length	The **average** of the shortest path length between all possible node pairs in a network. Usually only defined for connected graphs.	Are the system's entities strongly interconnected? A shorter characteristic path length indicates that nodes in the network are more closely connected. These networks, where every entity is connected to every entity, are sometimes called “small-world” networks.	All care activities involved in the care process are well interconnected. It indicates interdependence of care actions throughout the entire patient care process.
Density	The **fraction** of the number of edges in the present network to the total number of possible edges of the networks, ranges between 0 and 1.	How many of the possible relationships are present in the system? In dense networks (density close to 1), all entities have close relationships with each other. Sparse networks (density close to 0) indicate that relationships between system entities are hard to build.	In dense care activity networks, the care time of one activity highly correlates with the care time of other single activities and sets of activities. In contrast, in sparse networks, multiple care activities must be involved to impact the whole care process.
Global clustering coefficient or Transitivity	**Ratio** of the triangles and the connected triples in the graph, ranges from 0 to 1. It measures the overall tendency of nodes within a network to build clusters focusing on how interconnected a node’s neighbors are (Wasserman and Faust, 1994b).	To what extent do network nodes group together overall? A high global clustering coefficient (close to 1) indicates that nodes in the network tend to build clusters, and many cluster nodes have connections to their neighbors.	Care activity networks with a high global clustering coefficient indicate robust patterns of correlated care-time allocation among care activities, suggesting consistent bundles across individuals rather than simple co-occurrence of practices.
Average clustering coefficient	The **average** overall nodes’ local clustering coefficients. It measures the overall tendency of nodes to build clusters (Watts and Strogatz, 1998).	To what extent do the single nodes within the network group together? It focuses on the local neighborhood. A high average clustering coefficient (close to 1) indicates that nodes in the network tend to build clusters, and many cluster nodes have connections to their neighbors. Low average clustering coefficient (close to 0) may indicate a more hierarchical or tree-like structure.	Care activity networks with a high average clustering coefficient indicate that local consistent patterns of care. In contrast, a low average clustering coefficient may indicate that some activities are more isolated, pointing to individualized care or less predictable correlation patterns.
Modularity	It measures the **strength** of the division of the network into clusters (or modules), where nodes within clusters have dense inter-connections but are sparsely connected to nodes outside of the cluster.	Are structural clusters present in the network? How much are the clusters separated from each other? Values higher than 0.3 generally indicate significant community structure.	Care activity networks with high modularity indicate several different subsets of related care activities corresponding to specific maternity care patterns.

### 2.2 Node and edge-level centrality metrics

**Fig. 2:**
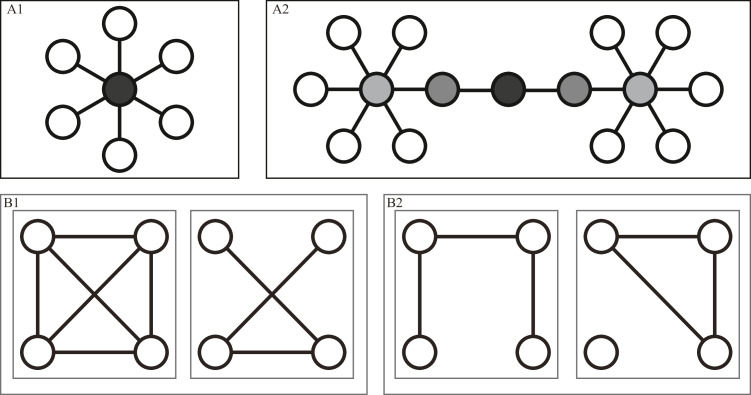
Different types of nodes within a network are assigned by their **centrality** (top), and types of networks are distinguished by different **densities** and **connectivities** (bottom). A1: Using shades of gray to assign **degree centrality**, the node in the middle shows a high node degree centrality. A2: The nodes’ **betweenness centrality** is assigned by shades of gray, showing the bridge node with the highest betweenness in the middle. B1: A dense (left) and a half-dense (right) network of *n* = 4 nodes where the maximum possible number of edges is $\frac{n*(n-1)}{2}$=6. B2: A connected (left) and a disconnected (right) network.

Different *centrality measures* of nodes or edges, such as those that explore structural aspects of networks, e.g. the importance of entities or relationships within the system, are known. The node degree centrality measures the number of direct links of a node to others, whereas the node and edge betweenness centrality measures how often the node or edge is involved in other relationships. For example, nodes that are often directly linked to other nodes (high node degree centrality) form the center of a group of related nodes, like the center of a star, and are often called hubs (Fig. 2, A1). In contrast, low-degree nodes with high betweenness centrality can signal a bridge within different sub-structures within the network (Fig. 2, A2). Furthermore, those edges with high betweenness centrality could indicate a relationship significant for the network, bridging or coordinating others. In the end, however, it is worth noting that although these measures seem intuitive and easy to estimate, there are no clear guidelines on how to interpret them in several fields and interpretation strongly relies on how the network was conducted (Bringmann et al., 2019; Borsboom et al., 2021).

### 2.3 Network-level structural metrics

Paths within networks are assigned by edges in a row within a network, and the path lengths measure *distances* between entities within networks. The length of a path between two nodes within a network is defined as the total number of edges on the path and illustrates how many entities are involved in a chain of relationships (Fig. 1). The shortest path between any two nodes is the minimum number of edges that must be passed when traveling between them. The diameter is a specific path within a network, representing the longest of all shortest paths. It therefore has a path length of the maximum shortest distance between any two nodes connected. For example, in Fig. 1 from all the shortest paths between all possible pairs of nodes, the longest one is between the nodes B and C and it has a length of 8.

Several other network properties summarize the global network structure. A network’s overall topology can be described, e.g. in terms of their density, connectivity and clustering effect. *Density*, which quantifies the number of edges in the present network in relation to the total number of possible edges given the number of nodes, is used to distinguish dense and sparse networks. Dense networks are those where all entities are closely related, and thus, many edges exist, while sparse networks are the opposite, in which only a few of all possible edges are present. A network's maximum number of edges is given by $\frac{n*(n-1)}{2}$, when n is the number of nodes. For example, in a network that includes 4 nodes with a maximum number of 6 edges, 3 existing edges would result in a half-dense network (Fig. 2 B1). Another distinction of networks is related to *connectivity*. Networks are defined as ‘connected’ if at least one path exists between every two nodes in the network (Fig. 2 B2), otherwise as ‘disconnected’. The characteristic path length is the average of all shortest path lengths in a network and measures the overall connectivity within a network, which is important for understanding its functionality. A further property of the network is the extent to which the overall network is separated into different single substructures. The *modularity* and *clustering coefficient* are two distinct concepts used to measure the strength of the cluster effect within networks; the first is how much the networks separate into different clusters, and the second is how much the entities of several clusters are related.

For our study, a network provides a *graphical representation* of the clinical care demand, where the nodes represent single care activities, and edges between them signify relationships (assessed by correlation) between pairs of care activities in the data. The network analysis can *identify hidden core elements of care characteristic* of the investigated population.

### 2.4 Study design, setting and sample

For this study, a retrospective explorative design was used. Care activity records of mothers from two maternity units of a tertiary-level hospital in Switzerland were used. We included all inpatient ‘postnatal’ cases of those units with a length of stay of one or more days in 2019. Inclusion required the absence of an explicit refusal of general consent. We excluded patients with diagnosis-related groups not referring to giving birth. Given the exploratory nature of this analysis, and the use of the network to visualize correlations in the data, no formal sample size calculation was performed a priori. However, the issue of sample size will be discussed in the limitation section.

### 2.5 Data

We used routine clinical care data extracted from the hospital’s electronic health record system. Records of care activities provide a list of documented care activities and care times needed per case per application (LEP® Nursing 3 Data). The LEP® Nursing 3 is a catalog of care activities used in this hospital and consists of roughly 700 care activities of which around 200–250 are utilized regularly. We extracted data for every care activity that was documented in the units, including information on when, by whom, to whom and how long an activity was applied (Table 2). For example, an entry within the care activity records could indicate that 2 min of a nurse's time (Care_Time) was required to check a mother's uterine position (Care_Activity) and was recorded at a specific time, here 20:15 on the 14th May 2019 (Date_Time), by one particular nurse (N_Provider).To ensure data quality and derive a robust dataset suitable for network analysis, the data pre-processing included the following steps: i) the validation of care activity times, ii) the identification of relevant dimensions, and iii) the aggregation of the multiple records of care activity times for each mother and care activity (detailed information of the pre-processing provided within Supplemental material 1).

**Table 2:** A simplified sample from a dataset of care activity records: each row shows for each individual (Patient_ID) when (Date_Time) and how long (Care_Time in minutes) a care activity (Care_Activity)) was applied and how many nurses/midwives (N_Provider) were needed.

Patient_ID	Care_Activity	Date_Time	N_Provider	Care_Time
P_00001	Conduct an admission discussion	2019–03–22 08:00:00	1	15
P_00001	Supporting breastfeeding	2019–04–22 08:00:00	1	9
…				
P_00002	Conduct an admission discussion	2019–05–14 08:00:00	1	5
P_00002	Monitoring the state of the uterus	2019–05–14 20:15:00	1	2
….				

### 2.6 Analysis

The network structure was derived using a partial correlation network approach. From the aggregated values of care time for each case and activity, derived by summing the total care times of each mother and care activity over the whole hospital stay, Pearson’s correlation was estimated for each pair of care activities to assess the weights of the network edges. In this context, the correlation reflects the extent to which the total time spent on two different care activities during a mother’s hospital stay tends to vary together across all mothers. Here, a correlation, for instance, describes whether mothers who received a higher amount of care time in one activity also tended to receive a higher amount of care time in another activity during their hospital stay. Additionally, to estimate a network based on partial correlations that accounts for indirect associations between variables, the graphical lasso method (Friedman, Hastie and Tibshirani, 2008) was used. An L1-penalization (λ = 0.05) was applied as a regularization technique to obtain a sparse network by removing weak edges. In practical terms, this means that no edge is drawn between two activities if their association is weak or can be explained by other activities in the network. The resulting network therefore highlights only those relationships that remain important when all activities are considered together. This resulted in a weighted undirected graph representation, where we further only considered edges with absolute correlation ≥ 0.15. We set the threshold to 0.15 to capture a network structure that includes care activity relationships with moderate absolute correlation values. The threshold affects visibility. If the threshold is set to only high correlations, only the standard care bundle would be visible. In contrast, if very low correlations were allowed, the bundle structure could be hidden because the network becomes too dense. Different edge, node, and network measures (according to Table 1) are calculated to interpret the network structure.

The overall network structure, representing the intricate system of mothers’ care demands in maternity care, can be used further to describe the complexity of care and identify hidden core characteristics of this population. For example, identifying hub activities (nodes with high node degree centrality) could highlight care activities that correlate with the application of a bundle of care activities and, therefore, are essential. In the following, we define care bundles as dense subgroups of care activities. Further, nodes with low degree and high betweenness centrality could hint at care activities at the boundary between two different sets of care activities and may signal a change in the type of care or level of complexity. Small distances between pairs of care activities within the network indicate that correlation between two care activities arises independently from relation to other care activities of the entire care process, especially when more time is needed for the first activity, which then increases (or decreases) the need for the second without being related to any other activity. Further, a dense care activity network would show that when more time is needed for applying one activity also more (or less) time is needed for the application of multiple other activities during the care process. Finally, measuring the tendency to separate the network into several clusters can help to explore different maternity care patterns.

The analysis was done on MacOS 15.3.2 using the programming language R, version 4.4.2 (R Core Team, 2024). The packages glasso (implementing the graphical lasso method), version 1.11 (Friedman, Hastie and Tibshirani, 2019) and igraph (for network illustration and analysis), version 2.1.4 (Csardi and Nepusz, 2006) were used.

### 2.7 Ethical considerations

The data for this study were obtained from a Swiss tertiary hospital. The dataset includes case, patient, and nurse/midwife records. All identifiers (case numbers, patient IDs, and staff IDs) were anonymized by the hospital before the researchers’ access, ensuring that no personally identifiable information was available to the research team. Routine anonymized data use of individuals was permitted unless general consent had been explicitly denied. The dataset is not publicly accessible; access was granted exclusively for this study under the hospital’s data protection and ethical approval procedures. The responsible ethical committee declared that the project is outside of the scope of the Human Research Act (Req-2022–01,153). This study was an observational analysis of routine data and no intervention was performed.

## 3 Results

### 3.1 Care activity data

Using the 2 346 cases, the dataset consists of 244 382 entries of documented care activities. The data indicates that 113 different care activities were applied to the mothers, and the total care time (over the whole hospital stay) needed for a single care activity ranged from 1 min to 340 min, while for 75 % of the activities, 5 min or fewer were needed. On average, applying a single care activity to a mother took 3.7 min (SD=3.9). The most frequent care activities (>99 % of cases) were ‘Measuring vital signs using a monitor’, ‘Monitoring the state of the uterus’ and also ‘Performing a breast examination’, which all belong to the standard postnatal care, while the most care time-consuming were ‘Supporting breastfeeding,’ ‘Providing guidance/instruction,’ ‘Performing a visit with a physician/treatment team,’ and ‘Conduct an admission discussion,’.

### 3.2 Network

The interpretation of the resulting network structure for this care demand data corresponds to the commonly used network characteristics introduced in the methods section and Table 1.

#### 3.2.1 Global network structure

**Fig. 3:**
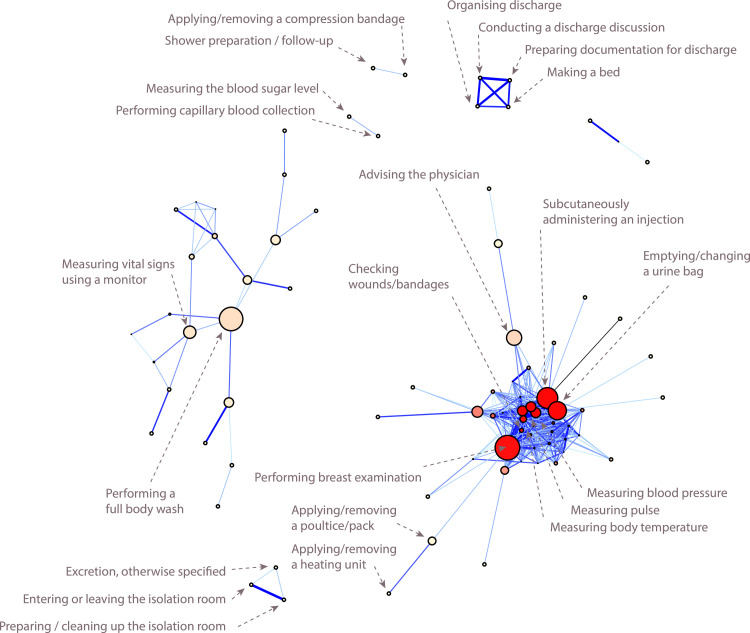
The Network based on the sample care data includes 84 care activities (nodes) - assigned as filled circles - and 489 connections (edges) - assigned as solid lines. The network shows two main subclusters of care activities. The node size represents the node’s betweenness centrality and the node’s color the node’s degree centrality of the activities in the network; line thickness and coloring represent the correlation strength of the two connected care activities.

Using the described correlation network approach resulted in a weighted undirected graph (Fig. 3) including 84 nodes, indicating the different care activities in maternity care, and 439 edges (a complete list of care activities within the network is provided as Supplemental material 2). For example, the two larger nodes at the center of the left cluster stand for the care activities ‘Performing a full body wash’ and ‘Measuring vital signs using a monitor’. The upper single 4-node substructure includes the 4 care activities (organizing discharge, conducting a discharge discussion, preparing documentation for discharge, and making the bed), indicating discharge management. Each node’s size represents its relative importance based on the nodes’ *betweenness* centrality and node’s color its relative importance based on the nodes’ *degrees* centrality of the activities in the network; line thickness and coloring represent correlation strength (lines indicating absolute correlation below 0.15 have been excluded). Hence, the greater (higher node betweenness centrality) and the redder (higher node degree centrality) the node, the more important and the more central within the network the corresponding care activity; the thicker and the bluer the lines, the more likely the time needed for the application of the two corresponding care activities is correlated. Two main clusters are prominent, and some smaller substructures exist within the network.

#### 3.2.2 Node characteristics

We measured within the network a median node degree centrality of 3.5 (Table 3), where twenty-four percent of the nodes have only one edge, and additionally 26 % have fewer than 4 edges. These nodes stand for care activities, which imply the application of only up to three other connected care activities, like e.g. ‘Applying/removing a poultice/pack’ (Fig. 3). In contrast, highly connected nodes - nodes with a high degree centrality - have a significant local influence on the surrounding nodes and stand for care activities or which the time spent providing care is strongly correlated with the time spent on many other care activities (see Fig. 2 A1). The nodes with the highest degree (red nodes with 30 or more direct connections) within the network include standard care actions (e.g. monitoring vital signs), as well as specialized care actions for mothers with a cesarean section (e.g. like administering an injection subcutaneously, emptying/changing urine bags and wound/bandage checks). These care activities, identified as high-degree centrality nodes, and the connected care activities can be seen as common parts of routine care in the overall maternity care process.

**Table 3:** Node-/Edge metrices obtained for the care activity network, presented as the median value [lower, upper quartiles], and with n specifying the number of nodes/edges.

Metrics	Value
**Nodes (*****n*****=84)**	
Node degree centrality	3.5 [2, 19.3]
Node betweenness centrality	1.96 [0, 20.8]
**Edges (*****n*****=439)**	
Edge betweenness centrality	3.2 [2.01, 5.35]

Considering not only direct connections, the small median node betweenness centrality of 1.96 (Table 3) and hence the small number of high betweenness nodes (4 biggest nodes with a betweenness centrality higher than 40) leads to the assumption of some existing critical intermediary or bottleneck care activities. Nodes bridging many other nodes across the whole network, indicated by a high betweenness degree, serve as a critical connector between different sets of care activities (see Fig. 2, A2). Those may serve as necessary care steps that link different phases or aspects of maternity care. For example, ‘Asking for physician advice’ could indicate postnatal complications, implying another type of and higher demand for care, including several specialized care activities.

‘Administering an injection subcutaneously’ and ‘Emptying/changing urine bags’ can be identified as hub activities, well-connected within their local neighborhood, and crucial in connecting different parts of the network (high node degree and betweenness centrality). This indicates that the time spent on one activity not only has a significant direct impact on the time spent on a bundle of other care activities but also has substantial indirect interaction between time spent on other care activities in various aspects of maternity care.

#### 3.2.3 Edge characteristics

Edges betweenness centrality ranges from 1 to 92 with a median centrality of 3.2 (Table 3), where only 8 % of the edges have a high (> 20) and 24 % have a low betweenness centrality (< 2). This combination suggests a structure with bundles of care activities, illustrating the several features of maternity care. The few edges with higher betweenness connect care activities of several maternity care features and mark key transitions in care provision. In contrast, the lowest betweenness edges are included in the small patterns not connected with the rest of the network, pointing to independent care bundles within the maternity care process. One example is the pattern indicating discharge management (Fig. 3), which consists of 4 care activities (organizing discharge, conducting a discharge discussion, preparing documentation for discharge, and making the bed).

#### 3.2.4 Network characteristics

With 84 nodes, the network's maximum possible number of edges is 3 486. By exhibiting only 439 edges, it has a low density value of 0.13 (Table 4), but it is still visually quite dense with an average node degree over 5. It is crucial to note that network density is influenced by the correlation threshold used to exclude edges, as the distribution of edge correlations determines the number of resulting nodes within the network.

**Table 4:** Network metrics obtained for the care activity network.

Metrics	Value
Diameter	6
Characteristic path length	2.15
Density	0.13
Global clustering coefficient	0.74
Average clustering coefficient	0.67
Modularity	0.2

The small characteristic path length of 2.1 indicates that, on average, the shortest path lengths within the network are relatively small (Table 4) and that many pairs of care activities are connected by short paths. The shortest path length between all pairs of two nodes ranges from 1 to 6. Thirty-one percent of the shortest paths have only one edge, and 70 % of them have a maximum of two edges, which shows that most existing connections in the network are relatively direct. This suggests a standardized care process involving many activities, which are essential for all mothers receiving maternity care. However, the presence of shortest paths longer than 2, particularly those with lengths of 5 or 6, indicates care activities for individualized care, possibly due to the occurrence of complications requiring different elements of specialized care.

Further analysis revealed a global clustering coefficient of 0.73 and an average clustering coefficient of 0.67, suggesting a strong tendency for nodes' neighbors to be interconnected within the network (Table 4). This results in the formation of dense subgroups of care activities or care bundles, indicating clusters of activities whose care-time allocations are strongly correlated during hospital stays.

The moderate level of modularity (value of 0.2) indicates that the network exhibits some degree of organization, with detectable modules where nodes within these modules are more interconnected than the overall network (Table 4). Initial visual inspection suggests two main clusters (Fig. 3), which we interpret as related to standardized versus individualized specialized care. However, all potential modules could be interpreted as care bundles but in our example, would not be clearly distinct or well-defined. This suggests that while identifiable groups of care activities exhibit correlated patterns in care time - either increasing or decreasing together - those care bundles cannot be defined directly as distinct care packages without further analysis.

## 4 Discussion

### 4.1 Introducing the network framework in health service research

In this article, we demonstrated the potential of the network framework for visualizing complex systems. Based on data on maternity care in a Swiss hospital, we explain how a population-based correlation network can be applied to explore the care complexity regarding the correlation between care activities within one Swiss hospital’s overall population of mothers after giving birth.

### 4.2 Insights from applying network analysis to maternity care data

The study's key finding is that network analysis of maternity care data reveals a standardized care process. Overall, the care activities network indicates a high level of interconnectedness among care activities and identifies two main care clusters, which point to standardized versus individualized specialized care. The network’s density and high interconnectivity suggest that care processes in the hospital are standardized by care bundles.

To further uncover the structure of the postnatal care processes, we used several network metrics aiming to identify key care components. For example, nodes with high degree centrality represent care activities whose care times are strongly correlated with those of many others. These activities are likely routine, standardized components of care, suggesting their potential to predict one bundle of care demand (e.g. monitoring vital signs). In contrast, nodes with high betweenness centrality act as bridges between different care bundles. These can be seen as indicators of changes in care demand from standard care to specialized care, probably due to complications. Their role in connecting otherwise separate parts of the network highlights their bridging effect in transitions between care levels.

Furthermore, metrics such as clustering coefficient and modularity provide insights into the presence of substructures or modules within the care network. A high average clustering coefficient or strong modularity may indicate care routines that operate semi-independently or bundled care packages. Taken together, these network properties help us understand which care activity bundles exist and how these are interconnected to be able to predict care demand in downstream analysis steps.

Our network analysis also indicates that some care activities (e.g. asking for a physician) are signs for changes in care (from standardized to specialized), since they were represented by high-degree and high-betweenness nodes in the care activity network. This care activity, ‘Asking for a physician’ could then be used as an indicator or signal for a change from standard postnatal care to the need for specialized care, which results in higher care time in the following days. Documented care activity data in the electronic health records could be routinely monitored to identify key care activities that may signal an increase in care time in subsequent days. They would thereby have the potential to inform unit managers about the upcoming change in care demand due to an increase in complexity.

### 4.3 Quantitative approaches to explore care demand and complexity

There is relatively little quantitative research on care demand in the field of postnatal inpatient. Birthrate Plus is a tool for classifying maternal cases into complexity categories. This category-based approach assigns specific amounts of care time based on the category aiming to ensure appropriate staffing in midwifery care (Ball, Washbrook and Royal College of Midwives, 2014; Ball and Washbrook, 2015). This instrument, although rather widely used, could benefit from a thorough validation process (Griffiths et al., 2023) and is not comparable to our time/tasks-based approach, which explores the entire spectrum of involved care activities. Electronic health records data documented by nurses provide a time- and task-based approach to quantifying care demand. For example, Cesare et al. (2025) describe care complexity in pediatric patients by analyzing the number and distribution of nursing diagnoses and nursing actions within medically defined groups. While this approach demonstrates that time-based activity data capture dimensions of care demand, it remains primarily list-based and does not examine how nursing activities are structurally interrelated within the care process. Similarly, the nursing activities score is widely used in intensive care to translate care complexity into workload by retrospectively recording and weighting 23 predefined nursing activities over a 24-hour period (Hoogendoorn et al., 2020; Reguera-Carrasco and Barrientos-Trigo, 2024). Although this instrument provides a standardized workload estimate, it aggregates activities into a single percentage value and relies on predefined weights, thereby simplifying the relational and processual nature of care. In contrast, our approach models care activities as a data-driven network derived from documented care, allowing the identification of structural interdependencies and discovering care bundles without relying on predefined categories or weighting schemes.

### 4.4 Future potential of network analysis for care demand research

In our study, we used postnatal mothers’ care activity records to analyze care demand and explore how care activities are interrelated through networks. This could be the basis for further comprehensive research, not only in maternity care but also in other care settings. Combining network-based clustering of care activities and individuals within a unit with a longitudinal analysis of care received may inform more effective adjustments to care supply.

In particular, using clustering methods on the care activity network derived from the documented care data, care activities could be grouped into meaningful “*care packages*” (defined here as care bundles). Applied to clinical care, such clustering identifies activities that show strong associations within the care process, thereby forming empirically derived distinct care bundles beyond traditional functional categories. The identification of these bundles is a data-driven methodological result of the network analysis and consequently needs additional validation from practitioners on meaningfulness and clinical relevance.

These validated care packages enable the development of a clear *care demand profile*. A profile may illustrate over time the amount of care time for each care package during the hospital stay. This future profile analysis would illustrate which care packages account for the largest proportion of documented care time at specific time points during the hospital stay and how maternity care demand shifts over time. This is furthermore a way to overcome the present static visualization of the overall care process. Unit managers could use this information about care time on each postnatal day to predict care time for the following care days for the mother and for the entire unit by summation of all mothers. In maternity care, such predictive profiling could support a timelier response to fluctuations in unit-level care demand and enable more efficient allocation of staff resources. By identifying which care packages are typically required at specific time points during the hospital stay, care delivery could be more closely aligned with mothers’ expected needs.

While we focused here on undirected networks, directed networks have also been widely used to describe complex systems, and infer potential causal relations, for example, in bioinformatics (Sachs et al., 2005) and psychology (Moffa et al., 2017). Such approaches may likewise help elucidate causal dependencies in care demand. Furthermore, in the context of cancer genomics, network-based clustering has been employed to stratify patients into those with similar mutational profiles (Kuipers et al., 2018; Bayer et al., 2025). Complementing directed causal models, undirected patient-similarity and brain connectivity networks in routine imaging, Electronic health records, and mental health data are already supporting precision medicine by revealing actionable patient subgroups with distinct comorbidity patterns, severity, and prognosis that standard feature-space clustering may miss (Cho et al., 2020; Martins et al., 2024; Yang et al., 2024). Correspondingly, care networks may hold additional information for clustering individuals based on their total received care time to identify *subgroups of individuals with similar care profiles*. One approach developed with undirected networks has been to derive individual-specific networks (Kuijjer et al., 2019; Li et al., 2023) and then using individuals' network contributions for clustering. This was demonstrated in a study on newborns’ microbiomes to uncover distinct subpopulations of newborns related to their diet and type of birth (Yousefi et al., 2023). Applying network-based clustering to individual-level care activity data could enable the identification of subgroups of mothers with similar care profiles, independent of their type of birth. Such a data-driven categorization may allow for a more precise alignment of care supply with patient-specific patterns of care needs.

Finally, combining both approaches, the identification of subgroups and the longitudinal presentation of main care packages over the hospital stay, would identify *different care profiles* that inform clinical managers to adjust care supply. If, for each care profile, the average proportion of care time for each package on each day of hospital care is estimated, this can be used to predict care demand in advance. Based on the number of care profile cases and the care day, the total care time for the next days in the unit can be predicted. Care demand peaks could be detected some days in advance and staff rostering adjusted accordingly.

Future studies could also integrate a network approach, plus e.g. *outcome measures*. For example, in postnatal care, examining associations between successful breastfeeding and distinct care profiles may help to identify care practices that are most conducive to positive maternal and infant outcomes. In addition, future exploratory work could relate documented indicators of patient need (such as nursing diagnoses or standardized assessments) to aggregated care activity profiles derived from network analysis, to examine whether patients with similar needs tend to receive similar patterns of care delivery.

### 4.5 Limitations of the study

The primary limitation of our study is the inadequate sample size for accurately estimating the identified network structure. Using Monte Carlo simulations (Constantin, Schuurman and Vermunt, 2023), it was determined that, with a sample size of approximately 2500, the weights of about 40 edges could be detected with 60 % sensitivity and 80 % specificity in a network with a density of 0.15. This suggests that regularization should have been stronger to reduce the number of edges (see Supplementary Material).

Furthermore, the care activity data derived from the LEP system represent documented care provided and were used as a proxy for care demand. It is important to note that recorded care activities reflect care that was delivered, which may differ from the actual underlying care demand. Unmet needs, documentation practices, staffing constraints, or organizational factors may influence the extent to which true care demand is reflected in recorded care activities. Since the data were collected routinely for documentation rather than research purposes, incomplete or missed entries during daily work under time pressure might lead to an underestimation of care demand. Consequently, the resulting network may not capture the full complexity of the care process. However, up to now, no other finely granulated and rich dataset of applied clinical care is available.

In this study, the total care time for each activity served as the basis for constructing the correlation network to illustrate maternity care complexity regarding correlations between care activities. While the correlation analysis of aggregated care times can provide important insights, it does not fully account for the joint occurrence of activities, as some tasks may overlap or take place simultaneously. A co-occurrence network analysis for pairs of care activities could offer an alternative perspective on the care network and uncover interdependencies that are not visible in a correlation-based approach. Future research should complement the current approach to provide a richer understanding of care processes.

Further, one limitation of constructing a static network is that it does not track changes over time. Therefore, with this first network analysis we cannot make statements about which care activities follow one another during the overall care process, nor can we determine whether at which specific day care activities are necessary. Dynamic networks would allow us to model how the structure of a care network and the relationships between care activities change over time. However, a comprehensive understanding of network methodology is necessary beforehand.

## 5 Conclusion

Overall, the network methodology is a promising tool for investigating complex systems within health services research and nursing care. It allows us to make complexity visible. Our study demonstrates that maternity care is a complex but standardized care process with individualized care elements that respond to postnatal complications. Network results can be used to identify signs of changes in care demand or different care profiles, which can inform managers in how to plan and adjust staff. By introducing network analysis to this example of maternity care, we hope other practical questions based on electronic health record data using network analysis will be raised.

## Funding

*This work was supported by the Schweizerische Eidgenossenschaft (Eidgenössische Stipendienkommission für ausländische Studierende) and the Stiftung Pflegewissenschaft Schweiz.*

## CRediT authorship contribution statement

**Diana Trutschel:** Writing – review & editing, Writing – original draft, Visualization, Validation, Project administration, Methodology, Investigation, Funding acquisition, Formal analysis, Data curation, Conceptualization. **Luisa Eggenschwiler:** Writing – review & editing, Writing – original draft, Validation, Investigation. **Niklaus Gygli:** Writing – review & editing, Writing – original draft, Validation, Investigation, Data curation. **Jack Kuipers:** Writing – review & editing, Writing – original draft, Supervision, Methodology. **Giusi Moffa:** Writing – review & editing, Writing – original draft, Supervision, Methodology. **Michael Simon:** Writing – review & editing, Writing – original draft, Supervision, Investigation, Conceptualization.

## Declaration of competing interest

The authors declare the following financial interests/personal relationships which may be considered as potential competing interests:

Diana Trutschel reports financial support was provided by Stiftung Pflegewissenschaft Schweiz. Diana Trutschel reports financial support was provided by Swiss Government. If there are other authors, they declare that they have no known competing financial interests or personal relationships that could have appeared to influence the work reported in this paper.

## Appendix Supplementary materials

Image, application 1

Image, application 2
