# Supplementary material for: Care demand networks in maternity care - an innovative approach exploring the complexity of care demands with routine data: Retrospective observational study
Source: Int J Nurs Stud Adv. 2026 Apr 16;10:100532. doi: 10.1016/j.ijnsa.2026.100532 (PMC13101619; doi:10.1016/j.ijnsa.2026.100532)
Supplement: Supplementary file 1 [file mmc1.pdf]

Supplemental Material 1

LEP data

To ensure data quality and derive a robust dataset suitable for network analysis, the data pre-processing included the following steps i) the validation of care activity times (LEP data), ii) the identification of relevant dimensions, and iii) the determination of an appropriate method to adapt longitudinally-measured care times to take full advantage of the available network analysis features.

Validation of the LEP data

LEP data was adjusted for several reasons: i.i) human documentation error leads to wrong LEP entries, and i.ii) there are synonyms for the same care activities in the LEP system; their documentation depends on the nurse's preference and leads to small sample sizes for some care activity items. Suspicious LEP entries were identified by their names (meaningful care activities for the entire population) or the exploration of the distribution of the care time (high or low values). Checks on the registered care time for each entry were controlled for implausible values, e.g., whether these were zero or excessively high. Based on a discussion with midwifery experts, such error entries were relabeled. For instance, for a case which had complications during post-natal care, 'Perform an information interview' was changed into 'Providing 1:1 support' for an entry with a high time indicating the need of care not the need of an interview. Furthermore, in these circumstances times were set to default times, or entries were deleted. For example, entries indicating 'measuring height' and 'measuring weight' - only carried out on babies and therefore wrongly assigned to the mothers – or 'providing CTG' - which is only performed pre-natal - were deleted. Additionally, some synonyms were discussed with midwifery experts and the local lead person for LEP data. Subsequently, different approaches were applied: 1) combine different care activity items into a meaningful new one (i.e., care activities 'Change the bed location' and 'Change rooms' were combined to the new 'Change the bed location/room'), 2) collapsing care activities to a related one (i.e., care activities 'Wash the hair' or 'Wash the back' were include into existing 'Perform partial body wash').

Identification of dimensions

After validation, the intended network analysis approach requires a dimensionality reduction (ii) to meaningful care activities to distinguish care activity structures. Therefore, all entries on 'Patient documentation' and 'Perform an information interview' were deleted, as they do not provide information for the entire sample, as they are applied routinely to all cases at admission and discharge within hospitals.

Provision of care demand feature

Finally, to prepare the data for the network analysis, for each care activity, the given LEP minutes were summed over the case's hospital stay to provide the total time during the hospital stay spent on each care activity for each woman as the final analysis feature.

Post-Hoc sample size Calculation

The R package ‘powerly’ and the work by[1] that it includes provide a Monte Carlo framework for estimating required sample sizes in network analysis. However, we were unable to apply it in an a priori manner in this study because it requires prior knowledge of key network properties, such as the expected number of nodes and network density. Once these properties are better established, they can be applied in future studies to guide more precise planning of data collection. Figure 1 shows that f with a sample of around 2,500 individuals a network of about 40 edges and a density of 0.15, the edges could be detected with 60% sensitivity and 80% specificity. Further simulations revealed that the recommended sample size needed to detect edges within a network of about 84 edges and a density of 0.15 is around 14500-15000 individuals. This corresponds to an additional data volume of 5 years of records.

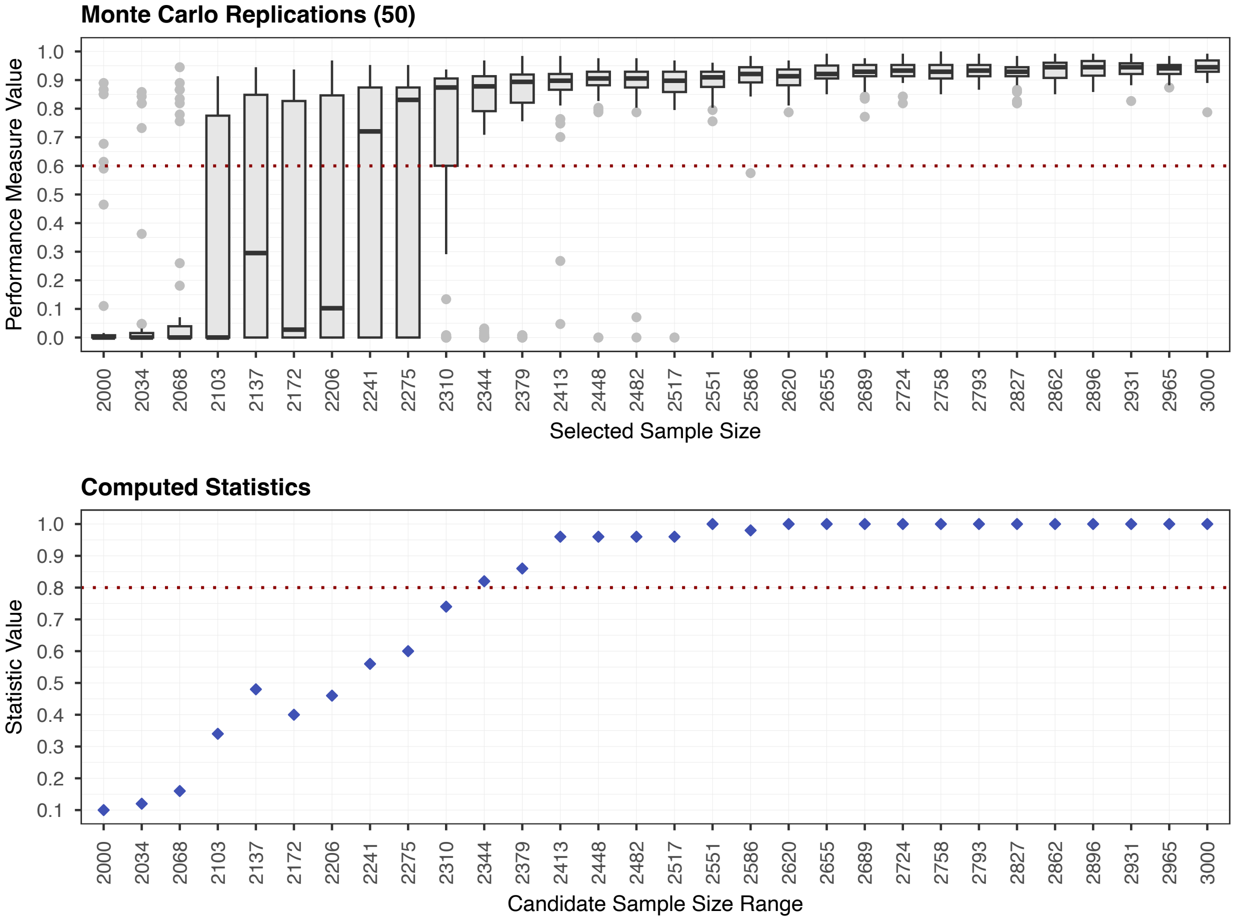

Figure 1: Sample Size calculation for care demand network using the R-package powerly (Version 1.8).

[1] Mihai A. Constantin, Noémi K. Schuurman, and Jeroen K. Vermunt, ‘A General Monte Carlo Method for Sample Size Analysis in the Context of Network Models’, *Psychological Methods* (US), published online 2023, doi:10.1037/met0000555.

Constantin, Mihai A., Noémi K. Schuurman, and Jeroen K. Vermunt, ‘A General Monte Carlo Method for Sample Size Analysis in the Context of Network Models’, *Psychological Methods* (US), published online 2023, doi:10.1037/met0000555
