# Supplementary material for: Care demand networks in maternity care - an innovative approach exploring the complexity of care demands with routine data: Retrospective observational study
Source: Int J Nurs Stud Adv. 2026 Apr 16;10:100532. doi: 10.1016/j.ijnsa.2026.100532 (PMC13101619; doi:10.1016/j.ijnsa.2026.100532)
Supplement: Supplementary file 2 [file mmc2.pdf]

Performing movement/mobilisation at bedside
Changing sanitary napkins
Providing / clearing away a beverage
Showering
Monitoring the state of the uterus
Administering an infusion with an additive
Providing support during vomiting
Administering an infusion
Subcutaneously administering an injection
Measuring the blood sugar level
Shower preparation / follow-up
Organising discharge
Providing guidance/instruction
Preparing and following up on personal care products
Washing the genital area
Measuring body temperature
Measuring fluids supplied
Performing a full body wash
Assisting with walking
Expressing breast milk
Entering or leaving the isolation room
Measuring the heartrate
Monitoring excretion
Inserting an indwelling cannula/catheter
Performing a partial body wash
Assisting in using the toilet
Emptying/changing a urine bag
Collecting a urine sample
Performing a visit with a physician / treatment team
Performing kangarooing
Checking wounds/bandages
Applying/removing a heating unit
Preparing/disposing of material/medication for self-care
Advising the physician
Measuring vital signs using a monitor
Preparing documentation for discharge
Performing an enema / bowel irrigation
Administering a blood transfusion
Preparing / breaking down an intensive care sleeping berth
Supporting breastfeeding
Measuring urine values / specific gravity
Reviewing case with physician / treatment team
Positioning/removing a rectal tube
Preparing/adjusting auxiliary aids
Making the bed
Attending to a wound
Providing advice on breastfeeding

Applying/removing a poultice/pack
Freshening up the patient
Monitoring pain
Performing breast examination
Orally administering medication
Preparing / cleaning up the isolation room
Performing a massage
Inserting ear plugs
Measuring blood pressure
Review, otherwise specified
Putting on / removing auxiliary aids
Performing inspection when starting a shift / taking charge
Dressing/undressing parts of the body
Configuring /setting up environment
Intravenously administering an injection
Performing capillary blood collection
Putting on/Removing compression stockings
Applying/removing a cooling unit
Implanting a gastric tube
Monitoring bleeding
Providing / clearing away a meal
Internal transportation
Inserting a urinary catheter
Setting up/breaking down a sleeping berth
Providing advice
Performing device/system-specific breakdown
Monitoring oxygen saturation
Assisting with a specific activity
Changing bed linen
Excretion, otherwise specified
Conducting a discharge discussion
Switching the sleeping berth or the room
Performing oral care
Positioning parts of the body
Mobilisation into or out of bed
Special monitoring
Placing/removing bedpan
